# On the Use of Caustic Potash, *in the First Stage of Carbuncle*

**Published:** 1839-12-21

**Authors:** Thomas F. Betton

**Affiliations:** Germantown, Pa.


### ON THE USE OF CAUSTIC POTASH,

*In the first stage of Carbuncle.*

BY THOMAS F. BETTON, M. D.

During the past summer, there came under my care a gentleman nearly eighty-six years of age, affected with carbuncle on the back of the head, just at the termination of the hairy scalp. They were two in number, close to each other, and had reached the second stage of the disease. The caustic potash was freely applied to each, and they pursued the usual course of the malady. During the progress of the cure, another made its appearance on the arm, just above the wrist, to which, also, in less than twenty-four hours after its discovery the potash was applied, and with the happiest results. The disease was arrested in its onset, the pain peculiar to carbuncle ceased, the inflammation progressed no further, and the sore healed as soon as an ulcer left by caustic potash heals under any other circumstances. Two more followed, one on each leg. Encouraged by the previous success, the potash was again resorted to, and with similar results.

A short time afterwards another case presented itself to my notice. A young woman, twenty-five years of age, asked my advice for what she supposed to be a boil, which she had been treating herself for some time. It proved to be carbuncle, situated on the shoulder: she then drew my attention to an inflamed spot on the calf of the leg, which, upon examination, appeared to be incipient anthrax. The caustic potash was applied to it, at first much against her inclination, but she expressed herself as much relieved in the course of an hour or two after its application. As in the first case, I had no reason to regret having used the remedy, although a severe one.

The practice, in the preceding cases, is so contrary to the generally received treatment of the first stage of anthrax, and also, I believe, as yet untried, that I have ventured, with much diffidence, to publish it. That it had the effect expected and intended, in the cases in which it was applied, there can be no doubt on my mind:—whether it is to be adopted or rejected as a mode of treatment, is to be proved by future experience. So convinced am I of its utility, that I would not hesitate to employ it again in the first suitable case that may occur to me.

The potash is to be applied over a surface proportionate to the extent of the inflammation. I have not found it necessary to apply it over a surface larger than could be covered by a dime. The ulcer left on the fall of the slough is perfectly healthy, and heals as rapidly as can be expected under similar circumstances.

I have neither the inclination nor the ability to extend the length of this paper with a disquisition on the modus operandi of the remedy; the facts are as above stated, and I leave them, in all confidence, to be confirmed or refuted by my medical brethren.

*Germantown, Pa.*, Dec. 12, 1839.

